# *n*-Propyl 2,3,4,6-tetra-*O*-acetyl-β-d-glucopyran­oside

**DOI:** 10.1107/S1600536812051495

**Published:** 2013-01-04

**Authors:** Bettina Mönch, Franziska Emmerling, Werner Kraus, Roland Becker, Irene Nehls

**Affiliations:** a BAM Federal Institute for Materials Research and Testing, Department of Analytical Chemistry, Reference Materials, Richard-Willstätter-Strasse 11, D-12489 Berlin, Germany

## Abstract

The title compound [systematic name: (2*R*,3*R*,4*S*,5*R*,6*R*)-2-(acet­oxy­meth­yl)-6-propoxytetra­hydro-2*H*-pyran-3,4,5-triyl triacetate], C_17_H_26_O_10_, was formed by a Koenigs–Knorr reaction of 2,3,4,6-tetra-*O*-acetyl-α-d-glucopyranosyl bromide and *n*-propanol. The central ring adopts a chair conformation. The crystal does not contain any significant inter­actions such as hydrogen bonds.

## Related literature

Metabolites of alcohol are important markers for previous alcohol consumption, see: Joya *et al.* (2012 ▶); Helander *et al.* (2012 ▶). For the investigation of the short-chain alkyl alcohol content in alcoholic beverages, see: Lachenmeier & Musshoff (2004 ▶). For the relevance of short-chain alkyl alcohol glucuronides as alcohol markers, see: Sticht & Käferstein (1999 ▶). For related synthesis, see: Baer & Abbas (1979 ▶).
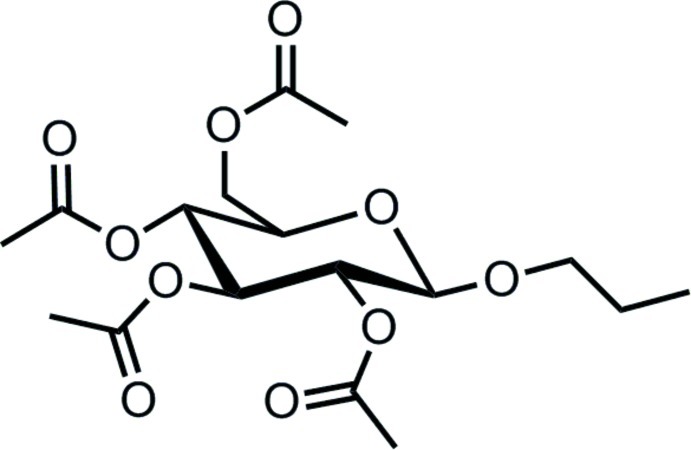


## Experimental

### 

#### Crystal data

C_17_H_26_O_10_
*M* *_r_* = 390.38
Orthorhombic, 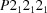

*a* = 7.0072 (11) Å
*b* = 15.215 (3) Å
*c* = 19.579 (3) Å
*V* = 2087.4 (6) Å^3^
*Z* = 4
Mo *K*α radiation
μ = 0.10 mm^−1^
*T* = 296 K
0.47 × 0.41 × 0.12 mm

#### Data collection

Bruker APEXII CCD diffractometer
Absorption correction: multi-scan (*SADABS*; Bruker, 2001 ▶) *T*_min_ = 0.152, *T*_max_ = 0.326
12995 measured reflections
2444 independent reflections
1286 reflections with *I* > 2σ(*I*)
*R*_int_ = 0.094

#### Refinement

*R*[*F* ^2^ > 2σ(*F* ^2^)] = 0.044
*wR*(*F* ^2^) = 0.122
*S* = 0.87
2444 reflections
244 parameters
H-atom parameters constrained
Δρ_max_ = 0.15 e Å^−3^
Δρ_min_ = −0.13 e Å^−3^

### 

Data collection: *APEX2* (Bruker, 2001 ▶); cell refinement: *SAINT* (Bruker, 2001 ▶); data reduction: *SAINT*; program(s) used to solve structure: *SHELXS97* (Sheldrick, 2008 ▶); program(s) used to refine structure: *SHELXL97* (Sheldrick, 2008 ▶); molecular graphics: *SHELXTL* (Sheldrick, 2008 ▶) and *ORTEPIII* (Burnett & Johnson,1996 ▶); software used to prepare material for publication: *SHELXTL*.

## Supplementary Material

Crystal structure: contains datablock(s) I, global. DOI: 10.1107/S1600536812051495/bt6871sup1.cif

Structure factors: contains datablock(s) I. DOI: 10.1107/S1600536812051495/bt6871Isup2.hkl

Supplementary material file. DOI: 10.1107/S1600536812051495/bt6871Isup3.mol

Additional supplementary materials:  crystallographic information; 3D view; checkCIF report

## Figures and Tables

**Fig. 1.**
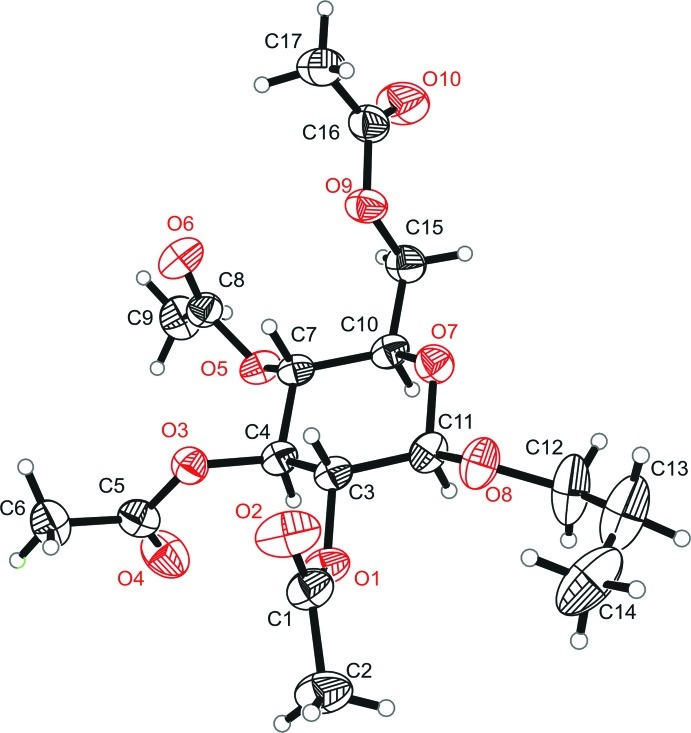
ORTEP representation of the title compound with atomic labeling shown with 30% probability displacement ellipsoids.

**Fig. 2.**
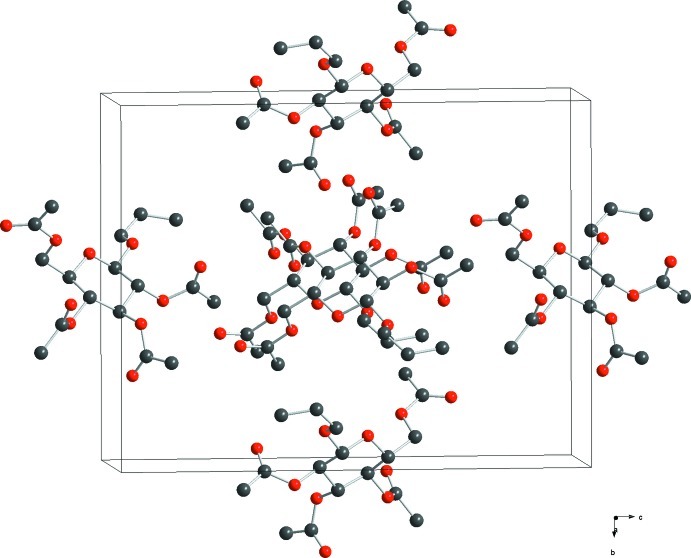
View of the unit cell of the title compound along the a axis.

## supplementary crystallographic information

### Comment

In recent years the determination of alcohol metabolites gained importance for screening previous alcohol consumption (Joya *et al.*, Helander *et al.*, 2012). Beside ethanol several short-chain alkyl alcohols, *e.g. n*-propanol, are found in alcoholic beverages as a result of fermentation process (Lachenmeier & Musshoff, 2004). The glucuronides of these so-called fusel alcohols are interesting markers for the consumption of alcohol (Sticht & Käferstein, 1999). So the analysis of these glucuronic metabolites, including their synthesis and full characterization is mandatory. The compound was formed by a Koenigs-Knorr-reaction of 2,3,4,6-tetra-*O*-acetyl-α-*D*-glucopyranosyl bromide and *n*-propanol (related synthesis Baer & Abbas, 1979) as an intermediate product towards synthesis of *n*-propyl-glucuronide.

The central ring has a chair conformation (Fig 1). One side chain of the molecule shows a small tendency to disorder, indicated in the *ORTEP* representation. This disorder leads to an unusual short contact C12—C13 (1.38 Å). The absolute configuration could not be defined confidently based on the single-crystal diffraction data. The isomeric purity of the title compound was confirmed by ^1^H-NMR.

### Experimental

*n*-Propyl 2,3,4,6-tetra-*O*-acetyl-β-*D*-glucopyranoside was synthesized by a Koenigs-Knorr-reaction of 2,3,4,6-tetra-*O*-acetyl-α-*D*-glucopyranosyl bromide and *n*-propanol. In order to obtain crystals suitable for single-crystal analysis, about 10 mg were dissolved in 2 ml *i*-propanol. Colourless crystals of the title compound were formed after 4 days of slow solvent evaporation at room temperature.

### Refinement

All H-atoms were positioned geometrically and refined using a riding model with d(C—H) = 0.93 Å, *U*_iso_=1.2*U*_eq_ (C) for aromatic 0.98 Å, *U*_iso_ = 1.2*U*_eq_ (C) for CH, 0.97 Å, *U*_iso_ = 1.2*U*_eq_ (C) for CH_2_, 0.96 Å, *U*_iso_ = 1.5*U*_eq_ (C) for CH_3_ hydrogen atoms. In the absence of significant anomalous dispersion effects 1815 Friedel pairs were merged. The absolute configuration has not been determined by anomalous-dispersion effects in diffraction measurements of the crystal. The conformation has been assigned due to an unchanging chiral centre in the synthetic procedure.

### Crystal data

**Table:**

C_17_H_26_O_10_	*F*(000) = 832
*M**_r_* = 390.38	*D*_x_ = 1.242 Mg m^−^^3^
Orthorhombic, *P*2_1_2_1_2_1_	Mo *K*α radiation, λ = 0.71073 Å
Hall symbol: P 2ac 2ab	Cell parameters from 1756 reflections
*a* = 7.0072 (11) Å	θ = 2.5–20.6°
*b* = 15.215 (3) Å	µ = 0.10 mm^−^^1^
*c* = 19.579 (3) Å	*T* = 296 K
*V* = 2087.4 (6) Å^3^	Block, colourless
*Z* = 4	0.47 × 0.41 × 0.12 mm

### Data collection

**Table:**

Bruker APEXII CCD diffractometer	2444 independent reflections
Radiation source: fine-focus sealed tube	1286 reflections with *I* > 2σ(*I*)
Graphite monochromator	*R*_int_ = 0.094
φ and ω scans	θ_max_ = 26.4°, θ_min_ = 1.7°
Absorption correction: multi-scan (*SADABS*; Bruker, 2001)	*h* = −8→8
*T*_min_ = 0.152, *T*_max_ = 0.326	*k* = −14→19
12995 measured reflections	*l* = −22→24

### Refinement

**Table:**

Refinement on *F*^2^	Primary atom site location: structure-invariant direct methods
Least-squares matrix: full	Secondary atom site location: difference Fourier map
*R*[*F*^2^ > 2σ(*F*^2^)] = 0.044	Hydrogen site location: inferred from neighbouring sites
*wR*(*F*^2^) = 0.122	H-atom parameters constrained
*S* = 0.87	*w* = 1/[σ^2^(*F*_o_^2^) + (0.0473*P*)^2^] where *P* = (*F*_o_^2^ + 2*F*_c_^2^)/3
2444 reflections	(Δ/σ)_max_ < 0.001
244 parameters	Δρ_max_ = 0.15 e Å^−^^3^
0 restraints	Δρ_min_ = −0.13 e Å^−^^3^

### Special details

**Table:**

Geometry. All e.s.d.'s (except the e.s.d. in the dihedral angle between two l.s. planes) are estimated using the full covariance matrix. The cell e.s.d.'s are taken into account individually in the estimation of e.s.d.'s in distances, angles and torsion angles; correlations between e.s.d.'s in cell parameters are only used when they are defined by crystal symmetry. An approximate (isotropic) treatment of cell e.s.d.'s is used for estimating e.s.d.'s involving l.s. planes.
Refinement. Refinement of *F*^2^ against ALL reflections. The weighted *R*-factor *wR* and goodness of fit *S* are based on *F*^2^, conventional *R*-factors *R* are based on *F*, with *F* set to zero for negative *F*^2^. The threshold expression of *F*^2^ > σ(*F*^2^) is used only for calculating *R*-factors(gt) *etc*. and is not relevant to the choice of reflections for refinement. *R*-factors based on *F*^2^ are statistically about twice as large as those based on *F*, and *R*- factors based on ALL data will be even larger.

### Fractional atomic coordinates and isotropic or equivalent isotropic displacement parameters (Å^2^)

**Table:**

	*x*	*y*	*z*	*U*_iso_*/*U*_eq_	
O1	−0.5821 (4)	−0.44666 (15)	0.11382 (11)	0.0723 (7)	
O2	−0.4530 (6)	−0.5422 (2)	0.18823 (14)	0.1102 (11)	
O3	−0.2268 (3)	−0.39673 (15)	0.05183 (11)	0.0658 (6)	
O4	−0.2904 (6)	−0.25348 (18)	0.03622 (15)	0.1102 (11)	
O5	−0.2593 (4)	−0.39896 (14)	−0.09808 (11)	0.0669 (7)	
O6	0.0304 (4)	−0.4603 (2)	−0.10821 (15)	0.0957 (9)	
O7	−0.5722 (4)	−0.57770 (15)	−0.04254 (12)	0.0729 (7)	
O8	−0.7407 (4)	−0.59780 (17)	0.05611 (13)	0.0857 (8)	
O9	−0.2672 (4)	−0.62575 (15)	−0.12723 (11)	0.0738 (7)	
O10	−0.2239 (5)	−0.6686 (2)	−0.23468 (15)	0.1215 (12)	
C1	−0.5591 (7)	−0.4835 (3)	0.17671 (19)	0.0767 (11)	
C2	−0.6884 (7)	−0.4384 (3)	0.22700 (18)	0.1032 (15)	
H2A	−0.6717	−0.4641	0.2714	0.155*	
H2B	−0.8187	−0.4453	0.2128	0.155*	
H2C	−0.6574	−0.3770	0.2289	0.155*	
C3	−0.4973 (5)	−0.4916 (2)	0.05674 (16)	0.0597 (9)	
H3A	−0.4081	−0.5364	0.0733	0.072*	
C4	−0.3908 (5)	−0.4247 (2)	0.01340 (16)	0.0591 (9)	
H4A	−0.4734	−0.3744	0.0034	0.071*	
C5	−0.1930 (7)	−0.3094 (3)	0.06182 (19)	0.0737 (11)	
C6	−0.0292 (7)	−0.2960 (3)	0.1081 (2)	0.0915 (14)	
H6A	−0.0080	−0.2342	0.1143	0.137*	
H6B	0.0827	−0.3224	0.0885	0.137*	
H6C	−0.0558	−0.3228	0.1514	0.137*	
C7	−0.3248 (5)	−0.4675 (2)	−0.05259 (15)	0.0560 (9)	
H7A	−0.2198	−0.5082	−0.0429	0.067*	
C8	−0.0781 (6)	−0.4022 (3)	−0.12245 (17)	0.0685 (10)	
C9	−0.0396 (7)	−0.3275 (3)	−0.16879 (19)	0.0945 (14)	
H9A	0.0885	−0.3317	−0.1859	0.142*	
H9B	−0.0545	−0.2733	−0.1443	0.142*	
H9C	−0.1277	−0.3290	−0.2063	0.142*	
C10	−0.4873 (5)	−0.5170 (2)	−0.08911 (17)	0.0639 (10)	
H10A	−0.5840	−0.4746	−0.1040	0.077*	
C11	−0.6544 (6)	−0.5349 (3)	0.01541 (18)	0.0699 (10)	
H11A	−0.7481	−0.4910	0.0007	0.084*	
C12	−0.9385 (8)	−0.6126 (4)	0.0406 (3)	0.135 (2)	
H12A	−1.0041	−0.5565	0.0400	0.162*	
H12B	−0.9480	−0.6377	−0.0048	0.162*	
C13	−1.0301 (9)	−0.6678 (5)	0.0862 (4)	0.162 (3)	
H13A	−0.9848	−0.7270	0.0775	0.195*	
H13B	−1.1649	−0.6670	0.0750	0.195*	
C14	−1.0134 (8)	−0.6520 (4)	0.1606 (3)	0.163 (3)	
H14A	−1.0800	−0.6974	0.1849	0.245*	
H14B	−1.0682	−0.5960	0.1716	0.245*	
H14C	−0.8812	−0.6525	0.1735	0.245*	
C15	−0.4194 (6)	−0.5683 (3)	−0.14971 (17)	0.0765 (11)	
H15A	−0.5234	−0.6027	−0.1686	0.092*	
H15B	−0.3731	−0.5288	−0.1848	0.092*	
C16	−0.1777 (7)	−0.6732 (3)	−0.1757 (2)	0.0798 (12)	
C17	−0.0204 (7)	−0.7261 (3)	−0.1465 (2)	0.0987 (15)	
H17A	0.0397	−0.7594	−0.1822	0.148*	
H17B	−0.0704	−0.7654	−0.1126	0.148*	
H17C	0.0718	−0.6877	−0.1258	0.148*	

### Atomic displacement parameters (Å^2^)

**Table:**

	*U*^11^	*U*^22^	*U*^33^	*U*^12^	*U*^13^	*U*^23^
O1	0.0767 (17)	0.0846 (17)	0.0556 (14)	0.0178 (15)	0.0109 (13)	0.0092 (12)
O2	0.130 (3)	0.124 (3)	0.0759 (18)	0.050 (3)	0.0144 (18)	0.0268 (17)
O3	0.0678 (16)	0.0658 (15)	0.0638 (14)	0.0028 (14)	−0.0051 (13)	0.0010 (12)
O4	0.149 (3)	0.0707 (18)	0.110 (2)	0.010 (2)	−0.029 (2)	−0.0002 (18)
O5	0.0688 (17)	0.0688 (16)	0.0631 (14)	0.0136 (14)	0.0140 (13)	0.0154 (12)
O6	0.0701 (19)	0.115 (2)	0.102 (2)	0.0170 (19)	0.0162 (16)	0.0149 (18)
O7	0.0765 (17)	0.0740 (16)	0.0683 (15)	−0.0015 (15)	0.0013 (14)	−0.0006 (13)
O8	0.0682 (18)	0.101 (2)	0.0875 (18)	−0.0126 (16)	−0.0038 (15)	0.0267 (16)
O9	0.0829 (19)	0.0780 (16)	0.0605 (14)	0.0182 (15)	−0.0064 (14)	−0.0084 (13)
O10	0.137 (3)	0.159 (3)	0.0689 (18)	0.028 (3)	−0.001 (2)	−0.029 (2)
C1	0.076 (3)	0.096 (3)	0.058 (2)	0.013 (3)	0.006 (2)	0.011 (2)
C2	0.114 (4)	0.135 (4)	0.061 (2)	0.024 (4)	0.019 (3)	0.004 (2)
C3	0.057 (2)	0.066 (2)	0.0557 (19)	0.0087 (18)	0.0038 (19)	0.0036 (18)
C4	0.059 (2)	0.061 (2)	0.058 (2)	0.0083 (19)	0.0021 (18)	0.0073 (17)
C5	0.091 (3)	0.068 (3)	0.062 (2)	−0.003 (2)	0.009 (2)	0.005 (2)
C6	0.101 (4)	0.096 (3)	0.078 (3)	−0.021 (3)	−0.007 (3)	−0.001 (2)
C7	0.060 (2)	0.057 (2)	0.0511 (18)	0.0119 (18)	0.0043 (18)	0.0055 (17)
C8	0.067 (3)	0.084 (3)	0.054 (2)	0.001 (3)	0.005 (2)	−0.001 (2)
C9	0.107 (4)	0.103 (3)	0.073 (2)	−0.023 (3)	0.015 (3)	0.013 (2)
C10	0.063 (2)	0.073 (2)	0.0561 (19)	0.015 (2)	−0.0013 (19)	0.0080 (19)
C11	0.063 (2)	0.078 (3)	0.069 (2)	0.002 (2)	0.002 (2)	0.016 (2)
C12	0.094 (4)	0.177 (6)	0.134 (4)	−0.059 (4)	−0.025 (4)	0.040 (4)
C13	0.084 (4)	0.224 (7)	0.179 (6)	−0.038 (5)	−0.009 (4)	0.065 (6)
C14	0.100 (5)	0.213 (7)	0.176 (6)	0.017 (5)	0.048 (5)	0.083 (6)
C15	0.084 (3)	0.087 (3)	0.059 (2)	0.011 (3)	−0.011 (2)	−0.0033 (19)
C16	0.086 (3)	0.087 (3)	0.067 (3)	−0.002 (3)	0.006 (2)	−0.013 (2)
C17	0.105 (4)	0.094 (3)	0.097 (3)	0.024 (3)	0.004 (3)	−0.015 (3)

### Geometric parameters (Å, º)

**Table:**

O1—C1	1.362 (4)	C6—H6B	0.9600
O1—C3	1.439 (4)	C6—H6C	0.9600
O2—C1	1.184 (5)	C7—C10	1.540 (5)
O3—C5	1.364 (4)	C7—H7A	0.9800
O3—C4	1.438 (4)	C8—C9	1.479 (5)
O4—C5	1.200 (5)	C9—H9A	0.9600
O5—C8	1.357 (5)	C9—H9B	0.9600
O5—C7	1.447 (3)	C9—H9C	0.9600
O6—C8	1.199 (4)	C10—C15	1.498 (5)
O7—C10	1.428 (4)	C10—H10A	0.9800
O7—C11	1.429 (4)	C11—H11A	0.9800
O8—C11	1.384 (4)	C12—C13	1.384 (7)
O8—C12	1.437 (5)	C12—H12A	0.9700
O9—C16	1.347 (4)	C12—H12B	0.9700
O9—C15	1.447 (4)	C13—C14	1.480 (7)
O10—C16	1.202 (4)	C13—H13A	0.9700
C1—C2	1.503 (6)	C13—H13B	0.9700
C2—H2A	0.9600	C14—H14A	0.9600
C2—H2B	0.9600	C14—H14B	0.9600
C2—H2C	0.9600	C14—H14C	0.9600
C3—C11	1.517 (5)	C15—H15A	0.9700
C3—C4	1.521 (5)	C15—H15B	0.9700
C3—H3A	0.9800	C16—C17	1.479 (6)
C4—C7	1.519 (4)	C17—H17A	0.9600
C4—H4A	0.9800	C17—H17B	0.9600
C5—C6	1.476 (6)	C17—H17C	0.9600
C6—H6A	0.9600		
			
C1—O1—C3	117.3 (3)	C8—C9—H9C	109.5
C5—O3—C4	120.2 (3)	H9A—C9—H9C	109.5
C8—O5—C7	119.2 (3)	H9B—C9—H9C	109.5
C10—O7—C11	112.3 (3)	O7—C10—C15	107.5 (3)
C11—O8—C12	114.1 (3)	O7—C10—C7	109.1 (3)
C16—O9—C15	116.9 (3)	C15—C10—C7	112.8 (3)
O2—C1—O1	123.8 (4)	O7—C10—H10A	109.1
O2—C1—C2	126.7 (4)	C15—C10—H10A	109.1
O1—C1—C2	109.5 (4)	C7—C10—H10A	109.1
C1—C2—H2A	109.5	O8—C11—O7	108.6 (3)
C1—C2—H2B	109.5	O8—C11—C3	108.1 (3)
H2A—C2—H2B	109.5	O7—C11—C3	109.2 (3)
C1—C2—H2C	109.5	O8—C11—H11A	110.3
H2A—C2—H2C	109.5	O7—C11—H11A	110.3
H2B—C2—H2C	109.5	C3—C11—H11A	110.3
O1—C3—C11	108.7 (3)	C13—C12—O8	114.0 (5)
O1—C3—C4	108.6 (3)	C13—C12—H12A	108.8
C11—C3—C4	110.4 (3)	O8—C12—H12A	108.8
O1—C3—H3A	109.7	C13—C12—H12B	108.8
C11—C3—H3A	109.7	O8—C12—H12B	108.8
C4—C3—H3A	109.7	H12A—C12—H12B	107.7
O3—C4—C7	109.2 (3)	C12—C13—C14	120.0 (6)
O3—C4—C3	107.3 (3)	C12—C13—H13A	107.3
C7—C4—C3	109.7 (3)	C14—C13—H13A	107.3
O3—C4—H4A	110.2	C12—C13—H13B	107.3
C7—C4—H4A	110.2	C14—C13—H13B	107.3
C3—C4—H4A	110.2	H13A—C13—H13B	106.9
O4—C5—O3	122.1 (4)	C13—C14—H14A	109.5
O4—C5—C6	126.9 (4)	C13—C14—H14B	109.5
O3—C5—C6	110.9 (4)	H14A—C14—H14B	109.5
C5—C6—H6A	109.5	C13—C14—H14C	109.5
C5—C6—H6B	109.5	H14A—C14—H14C	109.5
H6A—C6—H6B	109.5	H14B—C14—H14C	109.5
C5—C6—H6C	109.5	O9—C15—C10	107.9 (3)
H6A—C6—H6C	109.5	O9—C15—H15A	110.1
H6B—C6—H6C	109.5	C10—C15—H15A	110.1
O5—C7—C4	108.1 (2)	O9—C15—H15B	110.1
O5—C7—C10	107.5 (2)	C10—C15—H15B	110.1
C4—C7—C10	112.3 (3)	H15A—C15—H15B	108.4
O5—C7—H7A	109.6	O10—C16—O9	121.3 (4)
C4—C7—H7A	109.6	O10—C16—C17	127.1 (4)
C10—C7—H7A	109.6	O9—C16—C17	111.5 (3)
O6—C8—O5	122.5 (3)	C16—C17—H17A	109.5
O6—C8—C9	126.4 (4)	C16—C17—H17B	109.5
O5—C8—C9	111.0 (4)	H17A—C17—H17B	109.5
C8—C9—H9A	109.5	C16—C17—H17C	109.5
C8—C9—H9B	109.5	H17A—C17—H17C	109.5
H9A—C9—H9B	109.5	H17B—C17—H17C	109.5
